# Association between intravenous fluid administration and endothelial glycocalyx shedding in humans: a systematic review

**DOI:** 10.1186/s40635-024-00602-1

**Published:** 2024-02-26

**Authors:** Sara Sukudom, Lisa Smart, Stephen Macdonald

**Affiliations:** 1 Emergency Department, Royal Perth Hospital, PO Box 2213, Perth, WA 6000 Australia; 2 Emergency and Critical Care, Small Animal Specialist Hospital, Tuggerah, NSW Australia; 3 College of Science, Health, Engineering and Education, Murdoch University, Murdoch, WA Australia; 4 Centre for Clinical Research in Emergency Medicine, Harry Perkins Institute of Medical Research, Perth, WA Australia; 5 Medical School, University of Western Australia, Perth, WA Australia

**Keywords:** Sepsis, Critical care, Fluid therapy, Endothelium, Endothelial glycocalyx

## Abstract

**Introduction:**

Several studies have demonstrated associations between greater rate/volume of intravenous (IV) fluid administration and poorer clinical outcomes. One postulated mechanism for harm from exogenous fluids is shedding of the endothelial glycocalyx (EG).

**Methods:**

A systematic review using relevant search terms was performed using Medline, EMBASE and Cochrane databases from inception to October 2023. Included studies involved humans where the exposure was rate or volume of IV fluid administration and the outcome was EG shedding. The protocol was prospectively registered on PROSPERO: CRD42021275133.

**Results:**

The search yielded 450 articles, with 20 articles encompassing 1960 participants included in the review. Eight studies were randomized controlled clinical trials. Half of studies examined patients with sepsis and critical illness; the remainder examined perioperative patients or healthy subjects. Almost all reported blood measurements of soluble EG components; one study used in vivo video-microscopy to estimate EG thickness. Four of 10 sepsis studies, and 9 of 11 non-sepsis studies, found a positive relationship between IV fluid rate/volume and measures of EG shedding.

**Conclusions:**

A trend toward an association between IV fluid rate/volume and EG shedding was found in studies of stable patients, but was not consistently observed among studies of septic and critically ill patients.

**Supplementary Information:**

The online version contains supplementary material available at 10.1186/s40635-024-00602-1.

## Introduction

Intravenous (IV) isotonic crystalloid fluid resuscitation is a first-line intervention in the hemodynamic resuscitation of the patient with critical illness. There is increasing awareness of the potential adverse effects of IV fluid administration [1–3]. For example, in septic shock, preclinical and clinical studies have suggested that liberal IV fluid therapy may exacerbate the shock state and result in worse outcomes [4–7]. This has led to variation in practice [8], and a fluid-sparing management strategy is the subject of recently published and ongoing clinical trials in critical and perioperative care [9–12].

One mechanism by which IV fluid may cause harm is by inducing shedding of the endothelial glycocalyx (EG) [13]. The EG is a negatively charged mesh-like structure of proteoglycans and glycosaminoglycans, housed within an immobile plasma layer coating the luminal surface of the vascular endothelium [14]. It forms a barrier between endothelial cells and circulating blood, which is essential to the maintenance of hemostasis and vascular permeability in health. In critical illness such as septic shock, there is shedding of the EG [15]. Consequently, exposure of endothelial cells to circulating mediators of inflammation, both at the site of local tissue injury and more generally, is believed to result in leucocyte adhesion, fluid extravasation, and propagation of the systemic acute inflammatory response.

Evidence from preclinical models supports the hypothesis that rapid administration of exogenous IV fluid can exacerbate shedding of the EG [16–19]. Possible mechanisms include hemodilution, shear stress, and the effect of myocardial stretch releasing natriuretic peptides, which may activate matrix metalloproteinases at the EG surface [20–22]. The clinical implications of any adverse effects of rapid IV fluid administration on the endothelial surface in critical illness, in which endothelial shedding is likely already established, are uncertain.

Assessing real-time EG shedding in vivo is challenging [23], however, there are generally two methods for indirect assessment. Firstly, the in vivo thickness of the endothelium surface may be inferred by side-stream dark field microscopy, which estimates the difference between the edge of a capillary and that of a transiting erythrocyte, the so-called perfused boundary region (PBR) or cellular exclusion zone [24]. This assumes that EG responses in different vascular beds are similar to the visualized area, e.g., sublingual mucosa [25, 26] The second method involves measurement of soluble components of the EG circulating in blood. This may include cleaved ectodomains of proteoglycan molecules such as syndecan-1 (Syn-1), and glycosaminoglycan components such as heparan sulphate (HS) and hyaluronan. This method assumes that the measured biomarker concentrations correspond to the degree of EG shedding, and that this is uniformly distributed.

The aim of this review is to critically assess the clinical evidence supporting the hypothesis that exogenous IV fluids administered at higher volumes, and/or rates, is associated with increased shedding of the EG in people, compared to lower volumes and/or rates.

## Methods

This systematic review followed the Preferred Reporting Items for Systematic reviews and Meta-Analyses (PRISMA) guideline and was registered on the International Prospective Register of Systematic Reviews (PROSPERO: CRD42021275133). Two reviewers (SS and SM) independently performed the title and abstract screen, full-text review, data extraction, and risk of bias assessment. Discrepancies were resolved by consensus with involvement of a third reviewer (LS), if required.

### Search strategy

The Medline, Embase, and Cochrane databases were searched from inception to 19 November 2023 using the following keywords: “vascular endothelium”, “glycocalyx”, “endothelial surface layer”, “fluid therapy”, “intravenous fluid”, and “IV fluid” (see Additional file 1 for full search strategy). No language or other restrictions were applied. Authors’ own knowledge of the literature and snowballing of references were further used to identify relevant publications.

### Eligibility criteria

The following inclusion criteria were applied: (1) interventional studies; (2) prospective and retrospective observational studies of any duration; (3) studies that examined rate or volume of IV fluid administration of any type; (4) studies that assessed EG shedding by any method, including biomarker(s) and imaging.

The following exclusion criteria were applied: (1) in vitro and animal studies; (2) review articles, case series and reports, protocols, abstracts, letters, commentaries/editorials; (3) studies assessing fluid balance without assessing fluid regime via rate or volume.

### Data extraction and quality assessment

The following data items were collected using an extraction form as per the study protocol: year of publication, population (including baseline characteristics), sample size, study design, inclusion and exclusion criteria, intervention/exposure, outcome measure(s), principal findings, source of funding. Where required, missing or incomplete data were sought from the corresponding authors.

Quality of individual studies was independently assessed by two reviewers (SS and SM) using the Cochrane Risk of Bias tools, with involvement of a third reviewer (LS), if required [25, 26]. For randomized controlled trials, the RoB 2 tool was used; for observational studies, the ROBINS-I tool was used. For the included studies authored by LS and SM, SS performed this assessment independently.

### Statistical analysis

Descriptive statistics were used to summarize the findings of the review. Given the clinical heterogeneity of participants between studies, and the variety and non-standardized quantification of outcome measures, a pooled meta-analysis was not planned a priori and, instead, a narrative synthesis presented.

## Results

**Fig. 1:**
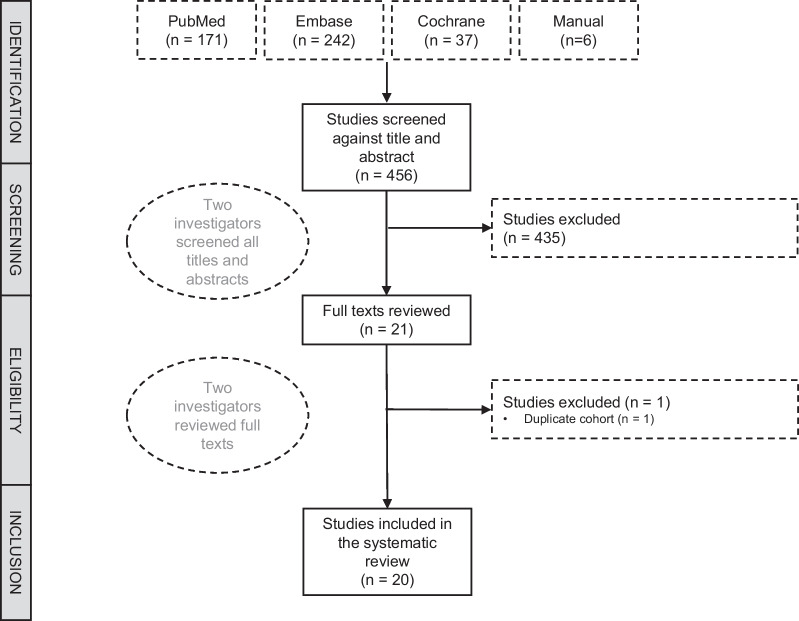
Flow diagram of study selection

The search yielded a total of 450 results, of which 17 duplicates were removed. An additional six records were identified by citation searching. Of the articles screened on the basis of title and abstract, 21 underwent full-text review, of which 20 studies including 1960 participants met our eligibility criteria [27–46]. Figure 1 summarizes the selection of articles.

**Fig. 2:**
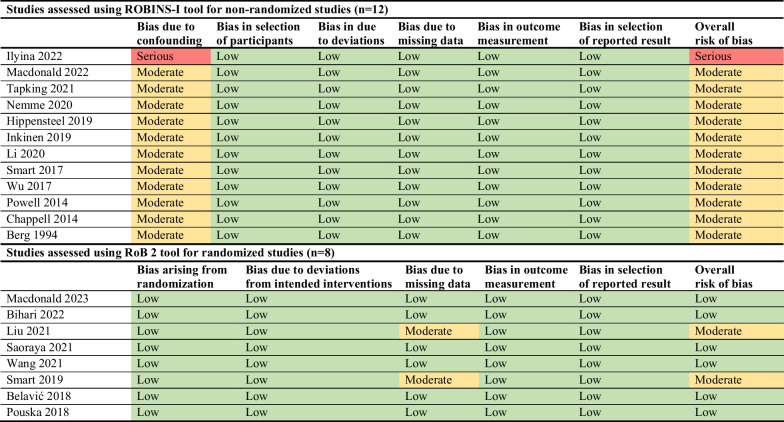
Risk of bias of included studies (*n* = 20)

The majority of included studies (*n* = 15; 75%) were published from 2018 onwards. Five papers originated from Australia, four from China, two from the United States, two from Germany, and one each in Croatia, the Czech Republic, Finland, Latvia, Russia, Sweden and Thailand. Eight studies used a randomized controlled trial design comparing fluid regimes by volume or infusion rate; seven were prospective observational studies examining 24-h fluid administration; five were non-randomized before-and-after trials involving an interventional fluid bolus. Assessment via the ROBINS-I and RoB-2 tools revealed that six studies were at low risk of bias, 13 studies were at moderate risk, and one was at serious risk (Fig. 2).

Half of the studies (*n* = 10) examined patients with suspected sepsis in the emergency department (ED) and/or intensive care unit (ICU) settings [27–36]. Most of the remainder (*n* = 8) were conducted in the perioperative setting [36–44], with one additional study including both sepsis and perioperative patients [36]. Two studies examined burns patients and healthy volunteers [45, 46]. All but one study included isotonic crystalloid fluid administration, although many studies stated the use of other fluid types: three studies included a combination of isotonic crystalloid and colloid (type not always specified), two studies included isotonic crystalloid and blood products, and five studies included isotonic crystalloid, colloid (type not always specified) and blood products. The use of synthetic colloid appeared minimal across most studies, although three studies in surgical patients included liberal use of hydroxyethyl starch solution [38, 42, 43]. Nine studies did not specifically clarify all fluid types administered during the study period.

Almost all studies reported measurement of EG components in blood as the method of assessment of shedding. In five studies, serial biomarker measures were adjusted for the effect of hemodilution using hematocrit or albumin [37, 38, 42, 44]. Syndecan concentration was measured as a marker of EG shedding in all but two studies. Eight studies measured HS concentration and eight measured hyaluronan concentration. Only one study employed sublingual side-stream dark field microscopy to measure EG thickness [36]. Table 1 summarizes the characteristics of included studies.

**Table 1:** Characteristics of included studies (*n* = 20)

References	Sample size, *n*	Country	Study design	Clinical setting	Intervention/exposure*	Primary outcome(s)	Secondary outcome(s)
Macdonald et al. [33]	95	Australia	Randomized controlled trial	Emergency/ICU; septic shock	Standard versus restricted IV fluid regime	Syn-1, Syn-4, HS, hyaluronan	Endothelial activation markers
Ilyina et al. [34]	18	Russia	Prospective before and after study	Emergency/ICU; septic shock	IV crystalloid bolus (7 mL/kg)	Syn-1, HS	Hemodynamic status, fluid response, LOS, mortality (28-day), severity of pulmonary edema
Macdonald et al. [32, 33]	86	Australia, United States	Prospective observational study	Emergency/ICU; septic shock	24-h IV fluid administration	Syn-1, Syn-4, hyaluronan	Endothelial activation markers, ANP, ICU admission, LOS, mortality (30-day)
Bihari et al. [44]	161	Australia	Randomized controlled trial	Major surgery; abdominal	Liberal versus restrictive IV crystalloid regime	Syn (subtype unspecified)	Post-operative complications, LOS, disability, mortality (90-day, 1-year)
Liu et al. [43]	54	China	Randomized controlled trial	Elective surgery; colorectal	Liberal versus restrictive IV fluid regime	Syn-1, HS, hyaluronan	ANP, LOS, post-operative recovery
Saoraya et al. [31]	96	Thailand	Randomized controlled trial	Emergency; sepsis	Standard versus limited rate IV fluid regime	Syn-1	Hemodynamic status, recovery, LOS, mortality (28-day, 90-day)
Tapking et al. [46]	39	Germany	Prospective observational study	Inpatient; burns	24-h IV fluid administration	Syn (subtype unspecified), HS	Mortality
Wang et al. [40]	80	China	Randomized controlled trial	Elective surgery; retroperitoneal tumor resection	Liberal versus restrictive IV crystalloid regime	Syn-1	Hemodynamic status, post-operative recovery/complications, mortality (1-year)
Nemme et al. [41]	26	Latvia	Prospective before and after study	Elective surgery; hysterectomy	IV crystalloid bolus (25 mL/kg)	Syn-1, HS	BNP
Hippensteel et al. [30]	201	United States	Prospective observational study	Emergency; septic shock	24-h IV fluid administration	Syn-1, HS	Endothelial activation markers, ANP
Smart et al. [35]	65	Australia	Randomized controlled trial	Emergency; sepsis	IV bolus 0.9 saline (10 mL/kg) versus 3% saline (5 mL/kg)	Syn-1, hyaluronan	Endothelial activation and inflammatory markers, hemodynamic status, 24-h fluid volumes, SOFA score, LOS
Inkinen et al. [29]	619	Finland	Prospective observational study	ICU; sepsis and post-operative patients	24-h IV fluid administration	Syn-1	Acute kidney injury, mortality (90-day)
Li et al. 2019	40	China	Two group prospective before and after study	Elective surgery; glioma resection; hepatectomy	IV colloid bolus (15 mL/kg)	Syn-1, HS	Hemodynamic status, cerebral metabolism markers
Pouska et al. [36]	66	Czech Republic	Randomized controlled trial	Emergency/ICU—septic shock patients; major spinal surgery	Fast versus slow rate IV crystalloid regime	Perfused boundary region	Hemodynamic status, fluid response
Belavić et al. [39]	90	Croatia	Randomized controlled trial	Elective surgery; laparoscopic cholecystectomy	Liberal versus restrictive IV crystalloid regime	Syn-1, hyaluronan	Kidney function, liver function, inflammatory markers
Smart et al. [28]	138	Australia	Prospective observational study	Emergency/ICU; sepsis	24-h IV fluid administration	Syn-1, Syn-4, hyaluronan	ICU admission, LOS, mortality (30-day)
Wu et al. [27]	26	China	Prospective observational study	Emergency/ICU—sepsis patients	24-h fluid balance	Syn-1	LOS, mortality (in-hospital)
Chappell et al. [38]	18	Germany	Unspecified	Elective surgery; general surgery	IV colloid bolus (20 mL/kg)	Syn-1, HS, hyaluronan	ANP
Powell et al. [37]	30	United States	Prospective before and after study	Elective surgery; cesarean section	IV crystalloid bolus (750 mL)	Syn-1, HS	ANP, hemodynamic status
Berg et al. [45]	12	Sweden	Prospective before and after study	Healthy volunteers	IV crystalloid bolus (1000 mL)	Hyaluronan	Hemodynamic status

*ANP* atrial natriuretic peptide, *BNP* brain natriuretic peptide, *HS* heparan sulfate, *IV* intravenous, *LOS* length-of-stay, *Syn* syndecan, *Syn-1* syndecan-1, *Syn-4* syndecan-4, *ICU* intensive care unit

^*^The word “fluid” is used where both isotonic crystalloid and colloid fluid types were included in the study

### Studies assessing patients with sepsis 

Ten studies examined sepsis populations, enrolling a total of 1360 participants. Four studies were randomized controlled trials [31, 33, 35, 36] and the remainder were prospective, observational studies [27–30, 32, 34]. The definition of sepsis varied. The Sepsis-3 criteria were used by three studies [31, 33, 34], Sepsis-2 or Systemic Inflammatory Response Syndrome (SIRS) criteria by five studies [27, 29, 30, 32, 35, 47], and in one, sepsis was not defined [36]. In general, participants were in late midlife, with the majority being male. A range of disease severities was represented. Two potential confounding variables, invasive ventilation and use of vasopressors, were variably reported among the studies. A summary of the sepsis studies is shown in Tables 2 and 3.

**Table 2:** Characteristics of participants in included sepsis studies (*n* = 10)

Reference	Intervention/exposure	Group size, *n*	Age, mean (SD)	Male, %	CCI, mean (SD)	SOFA score^a^, mean (SD)	Invasive ventilation, %	Vasopressor use, %	Mortality, % (days)
Macdonald et al. [33]	Standard fluid regimen group	49	65 (52,78)^b^	62	2 (1,4)^b^	5 (3,9)^b^	-	78	8 (90)
	Restricted fluid regimen group	46	66 (45,76)^b^	63	2 (0,5)^b^	5 (4,7)^b^	-	53	9 (90)
Ilyina [34]	Crystalloid fluid bolus (7 mL/kg)	18	55, 16	61	–	11	100	-	44 (28)
Macdonald [32, 33]	Total fluid administration	86	64, 18	60	3 (1,5)^b^	8, 4	43	64	13 (30)
Saoraya [31]	Standard fluid regimen group (30 mL/kg/h)	48	72, 16	62	5 (4,7)^b^	5 (2,6)^b^	42^c^	42^c^	25 (28); 31 (90)
	Limited fluid regimen group (10 mL/kg/h)	48	70, 18	60	5 (3,7)^b^	4 (2,5)^b^	23^c^	17^c^	17 (28); 19 (90)
Hippensteel [30] (A)	Total fluid administration	56	59, 15	54	3, 3	7, 4	–	–	9 (in-hospital)
Hippensteel [30] (B)	Total fluid administration	100	61, 3	66	4, 3	4, 4	–	–	11 (in-hospital)
Inkinen [29]	Total fluid administration	619	66 (55,75)^b^	64	–	8 (6,10)^b^	65	71	29 (90)
Pouska et al. [36]	Fast fluid bolus (5–10 min)	25	61, 18	43	–	10 (7,14)^b^	–	86	–
	Slow fluid bolus (25–30 min)	9	57, 19	67	–	8 (8,12)^b^	–	89	–
Smart [35]	10 ml/kg 0.9% NaCl	34	45 (39,52)^b^	58	0 (0,1)^b^	0 (0,1)^b^	–	3	3 (30)
	5 ml/kg 3% NaCl	36	41 (35,47)^b^	62	0 (0,1)^b^	0 (0,1)^b^	–	0	3 (30)
Smart [28]	Total fluid administration	86	59 (52,66)^b^	59	2 (1,4)^b^	4 (3,6)^b^	–	–	19 (30)
Wu [27]	Total fluid administration	15	65, 10	73	–	6, 3	–	–	27 (in-hospital)

*CCI* Charlson Co-morbidity Index, *SOFA* Sequential Organ Failure Assessment

^a^Measured at enrollment

^b^Value reported as median (interquartile range)

^c^Significant difference between intervention/exposure groups. Hippensteel (2019) study reports two separate cohorts

**Table 3:** Summary of findings in included sepsis studies (n = 10)

References	Intervention/exposure	Group size, *n*	Fluid volume (mL) at t = 24 h	Glycocalyx measure(s)	Summary of study findings
Macdonald et al. [33]	Standard fluid regime	49	3550 (2750,4410)^a,c,d^	Syn-1, Syn-4, HS, hyaluronan; *t* = 0 h, *t* = 3 h, *t* = 6 h, *t* = 24 h	No consistent relationship was found between fluid administration and glycocalyx markers. Syn-4 and hyaluronan showed greater or contrasting downward pattern in the restricted fluid group compared to standard. No differences were noted for Syn-1 or HS
	Restricted fluid regime	46	4360 (3350,5252)^a,c,d^		
Ilyina [34]	Crystalloid fluid bolus (7 mL/kg)	18	3560, 1870^b^	Syn-1, HS; *t* = 0 h, *t* = 2 h, *t* = 24 h	There was no significant change in Syn-1 or HS at any time point after fluid loading
Macdonald [32, 33]	Total fluid administration	86	4073, 2507^a^	Syn-1, Syn-4, hyaluronan; *t* = 0 h, *t* = 24 h	There was no significant relationship between cumulative fluid administration and glycocalyx markers, adjusted for age, sex, mean arterial pressure, lactate, CCI, SOFA score, infection source, and recruitment site
Saoraya [31]	Standard fluid rate (30 mL/kg/h)	49	3758 (1237,4975)^b,c,d^	Syn-1; *t* = 0 h, *t* = 6 h	There was no significant intergroup difference in Syn-1 over time, before and after adjusting for hemodynamic status and vasopressor use. There was no significant intra-group change in Syn-1 during the intervention
	Limited fluid rate (10 mL/kg/h)	49	2896 (1520,4535)^b,c,d^		
Hippensteel [30]	Total fluid administration	56	–	Syn-1, HS; *t* = 0 h, *t* = 6 h	Heparan sulfate concentration was significantly associated with cumulative fluid administration, after adjusting for age and sepsis severity (*p* = 0.047)
Hippensteel [30]	Total fluid administration	100	1681, 1801^a^	HS; *t* = 0 h, *t* = 24 h	Each 1L of administered fluid over 24 h was associated with a 200 ng/mL increase in heparan sulfate, after adjusting for age and sepsis severity (*p* = 0.006)
Inkinen [29]	Total fluid administration	619	5928 (4152,8712)^a,c^	Syn-1; *t* = 0 h, *t* = 24 h, *t* = 120 h	There was no significant relationship between Syn-1 and tertile of cumulative fluid administration
Pouska et al. [36]	Fast fluid bolus	25	–	PBR; *t* = 0 h, *t* = 1 h, *t* = 2 h	There were no significant intergroup differences at any time point after fluid bolus. No significant temporal change in PBR was observed in the general cohort
	Slow fluid bolus	9	–		
Smart [35]	10 ml/kg 0.9% NaCl		2000 (1000, 2500)^a,c^	Syn-1, hyaluronan; *t* = 0 h, *t* = 1 h, *t* = 3 h, *t* = 24 h	Significant fold-change in Syn-1 T0 to T1 in isotonic group corresponding with larger fluid volume in this period. No overall difference in biomarkers or fluid volume at 24 h. Low illness severity and majority patients had simple infection not sepsis
	5 ml/kg 3% NaCl		1500 (500–2500)^a,c^		
Smart [28]	Total fluid administration	86	3701 (2225,4750)^a,c^	Syn-1, Syn-4, hyaluronan; *t* = 0 h, *t* = 1–2 h, *t* = 3–5 h, *t* = 12–24 h	Each 1L of administered fluid was associated with a 22% increase in hyaluronan at *t* = 0 h, adjusted for infection severity and cytokine response (*p* = 0.001). This remained significant at *t* = 1–2 h and *t* = 3–5, but not at t = 12–24. No associations were noted for Syn-1 or Syn-4
Wu [27]	Total fluid administration	15	1616, 1113^b^	Syn-1; *t* = 0 h, *t* = 3 h, *t* = 6 h, *t* = 24 h, *t* = 48 h	Syn-1 levels were highly correlated with corresponding fluid balance at each of the four time points (*p* = 0.026)

*CCI* Charlson Co-morbidity Index, *HS* heparan sulfate, *PBR* perfused boundary region, *SOFA* Sequential Organ Failure Assessment, *Syn* syndecan, *Syn-1* syndecan-1, *Syn-4* syndecan-4

^a^Fluid volume reported as total fluid administered

^b^Fluid volume reported as fluid balance at t = 24 h

^c^Value reported as median (interquartile range)

^d^Significant difference between intervention/exposure groups

Overall, six of the 10 studies concluded no significant relationship between IV fluid administration and measured biomarkers of EG shedding in patients with sepsis or shock (Table 3). Of the nine studies that measured Syn-1 levels, only two found an association with administered fluid volume; one reported a positive correlation with fluid balance [27], and one reported a negative association with volume of fluid [35]. Hyaluronan was reported in six and HS in four studies, yielding mixed results for both biomarkers. All three studies that recruited a non-sepsis comparator group found higher levels of circulating EG markers in people with sepsis, compared to other groups [27, 28, 30]. EG shedding had either a neutral or positive relationship with poorer clinical outcomes. One study found higher Syn-1 levels in people admitted to the ICU, as opposed to those who were not [29]. Two studies found higher levels of Syn-1 [29] and HS [30] in non-survivors, compared to survivors. Conversely, one small study found no association between the measured EG biomarkers and ICU admission, mortality, or length-of-stay [27]. Smart et al. reported significant positive associations between Syn-1 and hyaluronan with Sequential Organ Failure Assessment (SOFA) score but not mortality [28]. EG biomarkers were not correlated with atrial natriuretic peptide (ANP) in either study that assessed this association [30, 32]. There was a moderate risk of confounding among the studies, owing to design and statistical factors.

Two randomized controlled trials examined rate of fluid administration and EG shedding, concluding no significant relationship between the two. Saoraya et al. randomized 98 patients presenting to a Thai ED with sepsis-induced hypoperfusion to receive either standard (30 mL/kg/h; *n* = 49) or limited (10 mL/kg/h; *n* = 49) rate fluid resuscitation [31]. The initial bolus was performed using Ringer’s lactate; for the ensuing six hours, fluid type was left to clinician discretion. At 24 h, the total volume of fluid administered was significantly higher in the standard rate group compared to the limited rate group. Syndecan-1 concentrations were not significantly different at the end of the 6-h intervention period, compared to baseline, for either group, nor were there any between-group differences, both before and after adjusting for baseline concentration, hemodynamic status and vasopressor use. Similarly, Pouska et al. randomized 16 patients admitted to a Czech ICU with sepsis to receive a fast (median 47 mL/minute; *n* = 7) or slow (median 11 mL/minute; *n* = 9) infusion of 5 mL/kg of isotonic crystalloid [36]. The PBR, as measured by sublingual side-stream dark field imaging, was not significantly different between groups at either 60 or 120 min after the infusion. Across both studies, there were no meaningful differences in parameters relevant to hemodynamic status between fast and slow fluid administration groups.

Two randomized controlled trials compared volumes of fluid administered. Macdonald et al. randomized ED patients with infection and hypotension to a fluid-restricted or usual care resuscitation regimen [33]. The usual care regime was associated with a slower decline in hyaluronan and syndecan-4 (Syn-4) concentrations compared to the fluid-restricted regime, but no effect was observed on Syn-1 or HS. Smart et al. randomized patients with sepsis to receive either 5 mL/kg of 3% saline or 10 mL/kg of 0.9% saline for initial fluid resuscitation in the ED [35]. Syndecan-1 and hyaluronan concentration increased over 24 h in both groups; however, only a minor difference in Syn-1 was detected between groups immediately after the fluid bolus.

Among the five observational studies, three reported a significant relationship between 24-h fluid volume, or fluid balance, and biomarkers of EG shedding [27, 28, 30]. Due to the design of the studies, it was not possible to control for fluid type; a mix of crystalloids, synthetic colloids and blood products was used as per local protocol. Hippensteel et al. included 156 sepsis patients across two centers in the United States, finding that cumulative IV fluid volume was positively associated with HS, after adjusting for age and sepsis severity [30]. Smart et al. found similar results among 86 patients recruited from an Australian ED, citing a direct association between cumulative IV fluid volume and hyaluronan concentration that was maintained after adjusting for sepsis severity and cytokine concentration; however, this did not apply to Syn-1 or Syn-4 [28]. Wu et al. found a positive association between Syn-1 and fluid balance among 15 participants [27]. By contrast, a multicentre study of 86 patients recruited in the ED concluded no significant relationship between IV fluid administration and Syn-1 or hyaluronan, adjusted for age, sex, mean arterial pressure, lactate, co-morbidities, sepsis severity, infection source, and recruitment site [32]. This finding was supported by a large Finnish study of 619 sepsis and non-septic ICU patients, which found no relationship between Syn-1 and tertile of IV fluid volume, although no adjustment for baseline confounders was made [29]. In addition, a Russian study involved administration of a 7 mL/kg IV crystalloid bolus to 18 patients with septic shock [34]. The authors concluded no significant change in Syn-1 or HS after fluid loading at any time point, compared to baseline, and no intergroup differences in biomarker concentration were detected between fluid responders and non-responders.

### Studies assessing non-sepsis patients

Nine articles examined perioperative and clinically stable populations, encompassing 600 participants. Five studies were randomized controlled trials [36, 39, 40, 43, 44], three were observational studies [37, 41, 42], and one had an unspecified method of group allocation [38]. Types of surgery included abdominal (general surgery; cholecystectomy; hepatectomy; colorectal; retroperitoneal tumor resection), pelvic (hysterectomy; cesarean section), and neurosurgical (brain; spinal). Only one study included non-elective surgery [37]. All surgical participants were subject to general anesthesia with IV fluid administered post-induction, except for one trial which examined the effects of fluid bolus prior to spinal anesthesia [37]. Most patients were relatively healthy with an American Society of Anesthesiologists (ASA) grade of I–II. A summary of these studies is shown in Tables 4 and 5.

**Table 4:** Characteristics of participants in included non-sepsis studies (n = 11)

Reference	Intervention/exposure	Group size, *n*	Age, mean (SD)	Male, %	ASA I–II, %	ASA III–IV, %	Duration of surgery, mean (SD)	Mortality, % (days)
Bihari [44]	Restrictive fluid regime at induction, intra-operative and post-operative	75	65, 13	32	34	66	132 (90,168)^a^	0 (90) 4 (365)
	Liberal fluid regime at induction, intra-operative and post-operative	86	67, 14	45	27	73	150 (102,204)^a^	0 (90) 2 (365)
Liu [43]	High-volume fluid (LR/HES) regime, post-induction	14	52, 8	50	100	0	181, 45	–
	Medium-volume fluid (LR/HES) regime, post-induction	17	51, 7	41	100	0	169, 49	–
	Low-volume fluid (LR/HES) regime, post-induction	16	50, 9	75	100	0	171, 54	–
Tapking [46]	Total fluid administration	39	45, 21	82	N/A	N/A	N/A	8 (in-hospital)
Wang [40]	Higher-volume fluid regime (LR) targeted at SVV 9%, intra-operative	40	49, 13	55	100	0	325, 78	5 (365)
	Lower-volume fluid regime (LR) targeted at SVV 14%, intra-operative	39	47, 13	49	100	0	349, 90	8 (365)
Nemme [41]	Rapid-rate crystalloid bolus (LR) at 25 mL/kg, post-induction	24	47, 5	0	–	0	–	–
Li [42]	Fluid loading (HES) at 15 mL/kg over 30 min, post-induction	40	47, 8	70	100	0	229, 77	–
Pouska [36]	Fast fluid bolus over 5–10 min, post-induction	25	60, 13	44	56	44	–	–
	Slow fluid bolus over 25–30 min, post-induction	25	62, 15	40	72	24	–	–
Belavić [39]	High-liberal fluid regime (LR) at 10-15 mL/kg/h intra-/postoperatively	30	57 (41,63)^a^	23	100	0	55 (50,70)^a,b^	–
	Low-liberal fluid regime (LR) at 5 mL/kg/h intra-/postoperatively	30	61 (41,70)^a^	30	100	0	58 (50,81)^a,b^	–
	Restrictive fluid regime (LR) at 1 mL/kg/h intra-/postoperatively	30	48 (40,62)^a,b^	27	100	0	58 (50,67)^a,b^	–
Chappell [38]	Fluid loading (HES) at 20 mL/kg over 15 min, post-induction	9	57, 4	–	–	–	–	–
	Acute normovolemic hemodilution (HES) at 60 mL/min, post-induction	9	55, 2	–	–	–	–	–
Powell [37]	Fluid bolus 750 mL (warmed LR) over 15 min, pre-induction	29	–	–	–	–	–	–
Berg [45]	Fluid bolus 1000 mL (LR) over 40 min	12	26, 4	67	N/A	N/A	–	–

*ASA* American Society of Anesthesiologists physical status classification, *LR* lactated Ringer’s solution, *HES* hydroxyethyl starch, *SVV* stroke volume variation

^a^Value reported as median (interquartile range)

^b^Significant difference between intervention/exposure groups

**Table 5:** Summary of findings in included non-sepsis studies (n = 11)

Reference	Intervention/exposure	Group size, *n*	Total fluid volume administered (mL)	Glycocalyx measure(s)	Summary of study findings
Bihari [44]	Restrictive fluid regime	75	3350 (2720, 4000)^a,b^ (*t* = 24 h)	Syn; intra-operative, post-operative, *t* = 24 h	There was no significant intergroup difference in Syn at baseline or over time through Day 1–Day 3 post-surgery
	Liberal fluid regime	86	6053 (5382, 7425)^a,b^ (*t* = 24 h)		
Liu [43]	High-volume fluid regime	16	–	Syn-1, HS, hyaluronan; pre-induction, post-induction, post-operative	Colloid administration and urine output were higher in the high-volume group; conversely, crystalloid administration was higher in the low-volume group. At the post-operative time point, Syn-1 and HS were higher in the high-volume group than medium- and low-volume groups
	Medium-volume fluid regime	17	–		
	Low-volume fluid regime	18	–		
Tapking [46]	Total fluid administration	39	–	Syn-1, HS; *t* = 0 h, *t* = 8 h, *t* = 24 h, *t* = 48 h	Syn-1 was significantly associated with total fluid volume administered at *t* = 8 h after burn (*p* < .05). A significant linear effect was observed of burn size and cumulative fluid administration in first 24 h on Syn-1 at *t* = 48 h (*p* = .011)
Wang [40]	High-volume fluid regime	40	4191, 1425^b^ (intra-operative)	Syn-1; *t* = 0 h, *t* = 1 h, *t* = 4 h, *t* = 24 h, *t* = 72 h	Syn-1 increased in both fluid regimen groups during surgery and declined postoperatively. No significant interaction between group and time for Syn-1
	Low-volume fluid regime	39	3284, 1145^b^ (intra-operative)		
Nemme [41]	Rapid-rate fluid bolus	24	–	Syn-1, HS; *t* = 0 h, *t* = 0.5 h, *t* = 1 h, *t* = 1.5 h, *t* = 2 h	There was overall minimal variation in Syn-1 and HS following fluid bolus, suggesting insignificant glycocalyx shedding. Plasma Syn-1 and HS increased between *t* = 1.5 h and *t* = 2 h. Urinary Syn-1 decreased during surgery
Li [42]	Fluid loading	40	–	Syn-1, HS; pre-induction, *t* = 0.5 h, post-operative	Syn-1 levels increased significantly after fluid load compared to baseline, after correcting for hematocrit HS levels (after hematocrit correction) showed no significant differences at any time point
Pouska [36]	Fast fluid bolus	25	–	PBR; baseline, *t* = 0 h, *t* = 20 min, *t* = 40 min, *t* = 1 h	There were no significant intergroup differences at any time point after fluid bolus. In the slow fluid bolus group, PBR increased after bolus at *t* = 0 h, then normalized
	Slow fluid bolus	25	–		
Belavić [39]	High-liberal fluid regime	30	5070 (4320–5775)^a,b^ (*t* = 6 h)	Syn-1, hyaluronan; *t* = 0 h, *t* = 6 h, post-operative	Syn-1 and hyaluronan levels were significantly lower in the restrictive fluid regime group compared to the high-liberal fluid group (*p* < 0.001). There were no significant differences between the restrictive and low-liberal fluid groups
	Low-liberal fluid regime	30	2370 (2190–2850)^a,b^ (*t* = 6 h)		
	Restrictive fluid regime	30	465 (420–575)^a,b^ (*t* = 6 h)		
Chappell [38]	Fluid loading	9	1326, 50	Syn-1, HS, hyaluronan; *t* = 0 h, *t* = 0.5 h	Hyaluronan and Syn-1 increased significantly in the fluid loading group at *t* = 0.5 h, while remaining unchanged in the hemodilution group (*p* < 0.05). There was minimal variation in HS overall. All values were corrected for albumin
	Acute normovolemic hemodilution	9	1267, 62 *drawn and replaced*		
Powell [37]	Fluid loading	29	750	Syn-1, HS; *t* = 0 h, post-bolus	Syn-1 and HS levels were both significantly increased following fluid loading (*p* < 0.05)
Berg [45]	Fluid bolus	12	1000	Hyaluronan; *t* = −0.5 h, *t* = 0 h, *t* = 10–70 min in 10-min intervals	Plasma hyaluronan increased significantly post-fluid bolus at *t* = 30 m, *t* = 40 m, *t* = 50 m, *t* = 60 m and *t* = 70 m (*p* < 0.05)

*HES* hydroxyethyl starch, *HS* heparan sulfate, *LR* lactated Ringer’s solution, *SVV* stroke volume variation, *Syn* syndecan, *Syn-1* syndecan-1, *Syn-4* syndecan-4

^a^Value reported as median (interquartile range)

^b^Significant difference between intervention/exposure groups

Overall, seven studies concluded a significant change in circulating EG markers in association with fluid administration [36–40, 42, 43], comprising either a positive relationship with fluid volume or a significant increase in biomarker concentrations after fluid administration. Eight studies measured syndecan levels; of these, three studies reported a positive association between Syn-1 and IV fluid volume [38, 39, 43], and three studies, an increase in Syn-1 after fluid administration, relative to baseline [37, 40, 42]. Two studies found no significant relationship [41, 44]. Five studies assessed HS, with one study showing increased HS after fluid loading compared to baseline [37], and the rest showing no associations with fluids [38, 41, 43]. Two out of three studies that assessed hyaluronan found either a positive association with IV fluid volume or an increase in concentration after fluid administration [38, 39]. Of the five studies that measured natriuretic peptide concentration, two studies demonstrated an increase in natriuretic peptide after fluid loading [38, 43], while two did not [37, 41], and one showed a positive association with fluid volume [39]. One study showed a positive correlation between ANP and EG biomarkers [43].

Among the five randomized controlled trials, four compared higher- versus lower-volume IV fluid regimes over 1–3 days [39, 40, 43, 44], while one compared fast versus slow rate of IV fluid bolus administration [36]. Two studies compared effects of a fixed fluid volume per hour based on patient body weight [39, 44], and two compared goal-directed stroke volume variation targets to guide ultimate fluid volume (SVV%) [40, 43]. A higher volume of fluid was used to maintain a lower SVV% target. Two trials, involving a total of 144 patients, found that patients that received a higher volume of fluids had higher concentrations of Syn-1, hyaluronan and ANP, compared to patients that received lower volumes [39, 43], while three trials found no differences in biomarker concentrations between groups [36, 40, 44]. Apart from minor differences found in hemodynamic parameters [36, 39, 43] and length of hospitalization between groups [43], no trials identified any major differences in clinical outcomes.

Among the four observational trials, three found a significant relationship between rapid fluid administration and EG biomarker levels. Li et al. performed fluid loading of 15 mL/kg hydroxyethyl starch (HES) over 30 min to 40 anesthetized patients, observing a significant rise in hematocrit-corrected Syn-1 compared to baseline, but no significant changes in HS [42]. The studies by Chappell et al. [38] and Powell et al. [37] found significant increases in some EG biomarkers, compared to baseline, following administration of HES and Ringer’s lactate, respectively. This change occurred prior to surgical incision. The participants in the latter study were not anesthetized and there was no significant change in brain natriuretic peptide (BNP). Notably, Chappell et al. found that hypervolemic fluid loading was associated with a significant increase in ANP, compared to baseline, whereas acute normovolemic hemodilution was not [38]. By contrast, Nemme et al. found minimal changes in Syn-1, HS, and BNP within an hour of rapidly administering 25 mL/kg Ringer’s lactate to 24 patients undergoing elective hysterectomy [41].

Two studies examined patient populations that were neither critically ill nor preoperative. The study by Tapking et al. recruited 39 patients admitted to the burns unit of a German hospital [46]. The authors concluded that Syn-1 was significantly associated with 24-h IV fluid volume, after adjusting for burn surface area. It is unclear whether this relationship was maintained at other time points. Likewise, an older study of 12 healthy volunteers receiving a 1000 mL bolus of Ringer’s lactate over 40 min found that this was associated with an increase in hyaluronan at each 10-min interval, up to 70 min post-bolus [45].

## Discussion

This systematic review identified 20 clinical studies that assessed the relationship between IV fluid administration and indicators of EG shedding in people. Across ten studies involving 1360 patients with sepsis or critical illness, it was found that EG markers were significantly elevated at baseline, but not consistently associated with rate or volume of IV fluid administration. Conversely, among nine studies of 600 stable and perioperative participants, there was a more consistent association between volume/rate of fluid administration and measures of EG shedding. These patterns were most evident when examining plasma Syn-1 concentration. Measurement of other biomarkers (e.g., Syn-4, hyaluronan and HS), as well as other methods of estimating EG thickness, were less frequently studied. The studies did not demonstrate a consistent relationship between rate/volume of fluid administration and natriuretic peptides, hemodynamic outcomes, or clinical outcomes such as length-of-stay and mortality.

To the best of our knowledge, this is the first review to synthesize evidence on rate/volume of IV fluid therapy and EG shedding from human clinical trials. Previous research, including a recent review by Hahn et al. has established a link between EG shedding and severity of critical illness such as sepsis, hemorrhagic shock, trauma and severe inflammatory states, with the aim of understanding how resuscitative strategies may better protect the EG [23, 47]. A review by Smart et al. explored evidence from preclinical and animal studies that suggested restrictive and/or slow fluid administration may be beneficial in these states, for instance, by reducing the deleterious early inflammatory response and decreasing subsequent vasopressor requirements [22]. It is proposed that restrictive regimens avoid fluid-mediated EG injury occurring via mechanisms such as release of EG-shedding matrix metalloproteinase in response to vascular stretching; oscillatory shear stress-induced activation of cathepsin L; facilitation of neutrophil-elastase-related EG shedding; and release of ANP causing EG degradation [48]. Such microcirculatory dysfunction may persist and lead to poorer outcomes despite initial improvement in macro-hemodynamic markers. The balance between micro- and macrocirculatory perfusion is termed hemodynamic coherence, and it is hypothesized—with supporting evidence from animal trials—that restrictive fluid regimes are better positioned to achieve this [50].

Despite this, our review found that clinical studies examining septic patients tended to observe no significant association between IV fluid rate/volume and EG shedding, while studies examining stable patients tended to observe a positive association. Several hypotheses may explain these findings. Firstly, variability may arise from differences in methodology between individual studies. Markers of EG shedding were measured at varying time points, with some studies comparing levels at baseline and within the first six hours, while others measured shedding at 24–48 h. With limitations on our knowledge of EG biomarker kinetics, it is unclear when plasma concentration peaks and when they are eliminated from the circulation; thus, there is currently no standardized method for measuring them. The same applies to clearance of natriuretic peptides, which were inconsistently associated with fluid administration in our review. Furthermore, it is understood that markers such as Syn-1 are renally excreted, meaning clearance is directly related to renal function [50, 51]. This may be significant when comparing critically ill and stable participants, given that the former are more likely to have acute renal impairment, in addition to higher baseline EG shedding. Reaching, and exceeding, steady-state clearance of EG biomarkers may affect measured levels in plasma during the studies. A similar issue arises when considering the effect of hemodilution on measured plasma concentration of biomarkers; in our review, only five studies of non-septic patients adjusted for hemoglobin, hematocrit, or albumin, acknowledging that any bias introduced by failure to adjust would be towards the null.

Physiological mechanisms may also explain the findings, although this remains speculative. Circulating levels of EG degradation products were generally elevated at baseline in the sepsis cohorts, consistent with previous literature demonstrating that EG shedding occurs during states of critical illness and inflammation [23]. In this case, a lack of association between IV fluid administration and markers of EG shedding may represent failure of the disrupted EG to respond to fluid therapy, regardless of rate or volume. This hypothesis was supported by findings from Pouska et al. which was the only study to measure EG shedding via PBR. They observed that PBR increased then normalized in the relatively healthy preoperative cohort, but remained at elevated baseline levels in the septic cohort [36]. Interestingly, a subgroup-analysis of the septic group found that PBR increased in hemodynamically fluid-responsive patients, but not among fluid non-responders [36]. It is possible that extensive EG damage precludes further appreciable shedding as measured by plasma biomarkers, and that this identifies patients at risk of fluid unresponsiveness, extravasation, and poorer clinical outcomes. Such patterns would underpin a rationale for monitoring microcirculatory changes as described by Xantus et al. allowing for a more personalized and proactive approach to fluid resuscitation [49]. More research is required, however, to understand which EG changes are likely to represent clinically meaningful information and to develop techniques to more reliably measure EG structure and function in clinically important vascular beds in humans.

Our findings should be interpreted in context of some limitations. Firstly, there was considerable heterogeneity between studies. Sources of inter-study variation included methods for measuring EG shedding, concurrent use of vasopressors, and adjustment for confounders. This resulted in findings that were not amenable to meta-analysis. Many studies were single-center studies with a small sample size, further limiting statistical robustness. Secondly, the mix of fluid type used during resuscitation was not standardized among sepsis studies; previous reviews investigating fluid type and EG shedding have suggested a potential theoretical benefit with naturally occurring colloids like albumin and fresh frozen plasma [50], however, clinical studies have not demonstrated benefit [51, 52]. Thirdly, there are limitations to using measured biomarkers in blood or urine as tools to quantify EG shedding. The molecules concerned are not specific to the EG but are widely expressed throughout the body; for example, Syn-1 is expressed in the liver, while hyaluronan is ubiquitously distributed in the body. Measuring PBR via side-stream dark field microscopy is more specific, although it is more user-dependent and less widely accessible, and only gives information about accessible vasculature rather than within vital organs [23].

Potential directions for future research include larger, randomized controlled trials evaluating the effect of restrictive and liberal fluid regimes on EG integrity among critically ill patients and healthy controls. Consideration of confounders arising from the clinical context, alongside closer analysis of EG changes during fluid administration and at follow-up, will be useful for investigating whether microcirculatory changes translate into meaningful clinical information.

## Conclusion

Despite preclinical studies indicating potential adverse effects of more liberal IV fluid therapy on EG integrity, there is limited evidence to support this in clinical practice within the limitations of currently available techniques. Most studies reporting a positive association between IV fluid volume/rate and EG shedding were in stable preoperative cohorts, rather than critically ill cohorts with higher baseline EG disruption. Further research is required address heterogeneity between trials and determine the clinical significance of these findings.

### Supplementary Information

**Additional file 1: Table S1.** Search strategies for databases run July 17th 2022. **Table S2.** Reference list of included articles.

## Data Availability

No original data were generated for this study.

## References

[1] Sakr Y, Rubatto Birri PN, Kotfis K, Nanchal R, Shah B, Kluge S (2017). Higher fluid balance increases the risk of death from sepsis: results from a large international audit. Crit Care Med.

[2] Silversides JA, Major E, Ferguson AJ, Mann EE, McAuley DF, Marshall JC (2017). Conservative fluid management or deresuscitation for patients with sepsis or acute respiratory distress syndrome following the resuscitation phase of critical illness: a systematic review and meta-analysis. Intensive Care Med.

[3] Malbrain M, Van Regenmortel N, Saugel B, De Tavernier B, Van Gaal PJ, Joannes-Boyau O (2018). Principles of fluid management and stewardship in septic shock: it is time to consider the four D's and the four phases of fluid therapy. Ann Intensive Care.

[4] Maitland KKS, Opoka RO, Engoru C, Olupot-Olupot P, Akech SO, Nyeko R, Mtove G, Reyburn H, Lang T, Brent B, Evans JA, Tibenderana JK, Crawley J, Russell EC, Levin M, Babiker AG, Gibb DM, on behalf of the FEAST trial group (2011). Mortality after fluid Olus in Africal children with severe infection. N Engl J Med.

[5] Maitland K, George EC, Evans JA, Kiguli S, Olupot-Olupot P, Akech SO (2013). Exploring mechanisms of excess mortality with early fluid resuscitation: insights from the FEAST trial. BMC Med.

[6] Andrews B, Semler MW, Muchemwa L, Kelly P, Lakhi S, Heimburger DC (2017). Effect of an early resuscitation protocol on in-hospital mortality among adults with sepsis and hypotension. JAMA.

[7] Byrne L, Obonyo NG, Diab SD, Dunster KR, Passmore MR, Boon AC (2018). Unintended consequences: fluid resuscitation worsens shock in an ovine model of endotoxemia. Am J Respir Crit Care Med.

[8] Keijzers G, Macdonald SP, Udy AA, Arendts G, Bailey M, Bellomo R (2019). The Australasian resuscitation in sepsis evaluation: fluid or vasopressors in emergency department sepsis, a multicentre observational study (ARISE FLUIDS observational study): Rationale, methods and analysis plan. Emerg Med Australas.

[9] Myles PS, Bellomo R, Corcoran T, Forbes A, Peyton P, Story D (2018). Restrictive versus liberal fluid therapy for major abdominal surgery. N Engl J Med.

[10] Meyhoff TS, Hjortrup PB, Wetterslev J, Sivapalan P, Laake JH, Cronhjort M (2022). Restriction of intravenous fluid in ICU patients with septic shock. N Engl J Med.

[11] Self WH, Semler MW, Bellomo R, Brown SM, deBoisblanc BP, Exline MC (2018). Liberal versus restrictive intravenous fluid therapy for early septic shock: rationale for a randomized trial. Ann Emerg Med.

[12] Macdonald SPJ, Keijzers G, Taylor DM, Kinnear F, Arendts G, Fatovich DM (2018). Restricted fluid resuscitation in suspected sepsis associated hypotension (REFRESH): a pilot randomised controlled trial. Intensive Care Med.

[13] Alphonsus CS, Rodseth RN (2014). The endothelial glycocalyx: a review of the vascular barrier. Anaesthesia.

[14] Reitsma S, Slaaf DW, Vink H, van Zandvoort MA, Oude Egbrink MG (2007). The endothelial glycocalyx: composition, functions, and visualization. Pflugers Arch.

[15] Jedlicka J, Becker BF, Chappell D (2020). Endothelial glycocalyx. Crit Care Clin.

[16] Torres Filho IP, Torres LN, Salgado C, Dubick MA (2016). Plasma syndecan-1 and heparan sulfate correlate with microvascular glycocalyx degradation in hemorrhaged rats after different resuscitation fluids. Am J Physiol Heart Circ Physiol.

[17] Torres LN, Sondeen JL, Ji L, Dubick MA, Torres FI (2013). Evaluation of resuscitation fluids on endothelial glycocalyx, venular blood flow, and coagulation function after hemorrhagic shock in rats. J Trauma Acute Care Surg.

[18] Ergin B, Guerci P, Uz Z, Westphal M, Ince Y, Hilty M (2020). Hemodilution causes glycocalyx shedding without affecting vascular endothelial barrier permeability in rats. J Clin Transl Res.

[19] Smart L, Boyd CJ, Claus MA, Bosio E, Hosgood G, Raisis A (2018). Large-volume crystalloid fluid is associated with increased hyaluronan shedding and inflammation in a canine hemorrhagic shock model. Inflammation.

[20] Bruegger D, Jacob M, Rehm M, Loetsch M, Welsch U, Conzen P (2005). Atrial natriuretic peptide induces shedding of endothelial glycocalyx in coronary vascular bed of guinea pig hearts. Am J Physiol Heart Circ Physiol.

[21] Bruegger D, Schwartz L, Chappell D, Jacob M, Rehm M, Vogeser M (2011). Release of atrial natriuretic peptide precedes shedding of the endothelial glycocalyx equally in patients undergoing on- and off-pump coronary artery bypass surgery. Basic Res Cardiol.

[22] Smart L, Hughes D (2021). The effects of resuscitative fluid therapy on the endothelial surface layer. Front Vet Sci.

[23] Hahn RG, Patel V, Dull RO (2021). Human glycocalyx shedding: systematic review and critical appraisal. Acta Anaesthesiol Scand.

[24] Massey MJ, Shapiro NI (2016). A guide to human in vivo microcirculatory flow image analysis. Crit Care.

[25] Aird WC (2007). Phenotypic heterogeneity of the endothelium: I. Structure, function, and mechanisms. Circ Res.

[26] Aird WC (2007). Phenotypic heterogeneity of the endothelium: II. Representative vascular beds. Circ Res.

[27] Wu X, Hu Z, Yuan H, Chen L, Li Y, Zhao C (2017). Fluid resuscitation and markers of glycocalyx degradation in severe sepsis. Open Med (Wars).

[28] Smart L, Macdonald SPJ, Burrows S, Bosio E, Arendts G, Fatovich DM (2017). Endothelial glycocalyx biomarkers increase in patients with infection during emergency department treatment. J Crit Care.

[29] Inkinen N, Pettila V, Lakkisto P, Kuitunen A, Jukarainen S, Bendel S (2019). Association of endothelial and glycocalyx injury biomarkers with fluid administration, development of acute kidney injury, and 90-day mortality: data from the FINNAKI observational study. Ann Intensive Care.

[30] Hippensteel JA, Uchimido R, Tyler PD, Burke RC, Han X, Zhang F (2019). Intravenous fluid resuscitation is associated with septic endothelial glycocalyx degradation. Crit Care.

[31] Saoraya J, Wongsamita L, Srisawat N, Musikatavorn K (2021). The effects of a limited infusion rate of fluid in the early resuscitation of sepsis on glycocalyx shedding measured by plasma syndecan-1: a randomized controlled trial. J Intensive Care.

[32] Macdonald S, Bosio E, Shapiro NI, Balmer L, Burrows S, Hibbs M (2022). No association between intravenous fluid volume and endothelial glycocalyx shedding in patients undergoing resuscitation for sepsis in the emergency department. Sci Rep.

[33] Macdonald S, Bosio E, Keijzers G, Burrows S, Hibbs M, O'Donoghue H et al.; REFRESH Trial Investigators. Effect of intravenous fluid volume on biomarkers of endothelial glycocalyx shedding and inflammation during initial resuscitation of sepsis. Intensive Care Med Exp. 2023;11(1):21. 10.1186/s40635-023-00508-4. 10.1186/s40635-023-00508-4 PMC10106534 37062769

[34] Ilyina Y, Fot E, Kuzkov V, Kirov M (2022). The glycocalyx shedding influences hemodynamic and metabolic response to fluid load in septic shock. Turk J Anaesthesiol Reanim.

[35] Smart L, Macdonald SPJ, Bosio E, Fatovich D, Neil C, Arendts G (2019). Bolus therapy with 3% hypertonic saline or 0.9% saline in emergency department patients with suspected sepsis: a pilot randomised controlled trial. J Crit Care.

[36] Pouska J, Tegl V, Astapenko D, Cerny V, Lehmann C, Benes J (2018). Impact of intravenous fluid challenge infusion time on macrocirculation and endothelial glycocalyx in surgical and critically Ill patients. Biomed Res Int.

[37] Powell MF, Mathru M, Brandon A, Patel R, Frolich MA (2014). Assessment of endothelial glycocalyx disruption in term parturients receiving a fluid bolus before spinal anesthesia: a prospective observational study. Int J Obstet Anesth.

[38] Chappell D, Bruegger D, Potzel J, Jacob M, Brettner F, Vogeser M (2014). Hypervolemia increases release of atrial natriuretic peptide and shedding of the endothelial glycocalyx. Crit Care.

[39] Belavic M, Sotosek Tokmadzic V, Fisic E, Brozovic Krijan A, Strikic N, Loncaric Katusin M (2018). The effect of various doses of infusion solutions on the endothelial glycocalyx layer in laparoscopic cholecystectomy patients. Minerva Anestesiol.

[40] Wang X, Duan Y, Gao Z, Gu J (2021). Effect of goal-directed fluid therapy on the shedding of the glycocalyx layer in retroperitoneal tumour resection. J Coll Physicians Surg Pak.

[41] Nemme J, Krizhanovskii C, Ntika S, Sabelnikovs O, Vanags I, Hahn RG (2020). Hypervolemia does not cause degradation of the endothelial glycocalyx layer during open hysterectomy performed under sevoflurane or propofol anesthesia. Acta Anaesthesiol Scand.

[42] Li X, Sun S, Wu G, Che X, Zhang J (2020). Effect of hydroxyethyl starch loading on glycocalyx shedding and cerebral metabolism during surgery. J Surg Res.

[43] Liu Y, Chen G, Gao J, Chi M, Mao M, Shi Y (2021). Effect of different levels of stroke volume variation on the endothelial glycocalyx of patients undergoing colorectal surgery: a randomized clinical trial. Exp Physiol.

[44] Bihari S, Dixon DL, Painter T, Myles P, Bersten AD (2021). Understanding restrictive versus liberal fluid therapy for major abdominal surgery trial results: did liberal fluids associate with increased endothelial injury markers?. Crit Care Explor.

[45] Berg S, Engman A, Hesselvik JF, Laurent TC (1994). Crystalloid infusion increases plasma hyaluronan. Crit Care Med.

[46] Tapking C, Hernekamp JF, Horter J, Kneser U, Haug V, Vogelpohl J (2021). Influence of burn severity on endothelial glycocalyx shedding following thermal trauma: a prospective observational study. Burns.

[47] Puskarich MA, Cornelius DC, Tharp J, Nandi U, Jones AE (2016). Plasma syndecan-1 levels identify a cohort of patients with severe sepsis at high risk for intubation after large-volume intravenous fluid resuscitation. J Crit Care.

[48] Cooper ES, Silverstein DC (2021). Fluid therapy and the microcirculation in health and critical illness. Front Vet Sci.

[49] Xantus G, Allen P, Kanizsai P (2021). Blind spot in sepsis management—tissue level changes in microcirculation. Physiol Int.

[50] Milford EM, Reade MC (2019). Resuscitation fluid choices to preserve the endothelial glycocalyx. Crit Care.

[51] Finfer S, Bellomo R, Boyce N, French J, Myburgh J, Norton R (2004). A comparison of albumin and saline for fluid resuscitation in the intensive care unit. N Engl J Med.

[52] Caironi P, Tognoni G, Masson S, Fumagalli R, Pesenti A, Romero M (2014). Albumin replacement in patients with severe sepsis or septic shock. N Engl J Med.

