# Supplementary material for: Association between intravenous fluid administration and endothelial glycocalyx shedding in humans: a systematic review
Source: Intensive Care Med Exp. 2024 Feb 26;12:16. doi: 10.1186/s40635-024-00602-1 (PMC10894789; doi:10.1186/s40635-024-00602-1)
Supplement: Supplementary file 1 — Additional file 1: Table S1. Search strategies for databases run July 17th 2022. Table S2. Reference list of included articles. [file 40635_2024_602_MOESM1_ESM.docx]

Additional Material

# Additional Table 1. Search strategies for databases run July 17^th^ 2022

| **Database** | **Access date** | **Search terms** | **Number of records identified** |
| --- | --- | --- | --- |
| MEDLINE | November 19^th^, 2023 | ("vascular endothelium" OR glycocalyx OR "endothelial surface layer") AND ("fluid therapy" OR "intravenous fluid*" OR "infusion fluid*" OR "IV fluid*") | 171 |
| EMBASE | November 19^th^, 2023 | ("vascular endothelium" OR glycocalyx OR "endothelial surface layer") AND ("fluid therapy" OR "intravenous fluid*" OR "infusion fluid*" OR "IV fluid*") | 242 |
| Cochrane | November 19^th^, 2023 | ("vascular endothelium" OR glycocalyx OR "endothelial surface layer") AND ("fluid therapy" OR "intravenous fluid*" OR "infusion fluid*" OR "IV fluid*") | 37 |

**Additional Table 2. Reference list of included articles**

| **ID** | **Reference** |
| --- | --- |
| 1 | Belavić M, Fišić E, Strikić N, Žunić J. The effect of various doses of infusion solutions on the endothelial glycocalyx layer in laparoscopic cholecystectomy patients. Minerva anestesiologica. 2018;84(9):1032-43. |
| 2 | Berg S, Engman A, Hesselvik JF, Laurent TC. Crystalloid infusion increases plasma hyaluronan. Critical care medicine. 1994;22(10):1563-7. |
| 3 | Bihari S, Dixon D-L, Painter T, Myles P, Bersten AD. Understanding Restrictive Versus Liberal Fluid Therapy for Major Abdominal Surgery Trial Results: Did Liberal Fluids Associate With Increased Endothelial Injury Markers? Critical care explorations. 2021;3(1). |
| 4 | Chappell D, Bruegger D, Potzel J, Jacob M, Brettner F, Vogeser M, et al. Hypervolemia increases release of atrial natriuretic peptide and shedding of the endothelial glycocalyx. Critical care. 2014;18(5):1-8. |
| 5 | Hippensteel JA, Uchimido R, Tyler PD, Burke RC, Han X, Zhang F, et al. Intravenous fluid resuscitation is associated with septic endothelial glycocalyx degradation. Critical care. 2019;23(1):1-10. |
| 6 | Ilyina Y, Fot E, Kuzkov V, Kirov M. The Glycocalyx Shedding Influences Hemodynamic and Metabolic Response to Fluid Load in Septic Shock. Turkish Journal of Anaesthesiology and Reanimation. 2022;50(2):94-100. |
| 7 | Inkinen N, Pettilä V, Lakkisto P, Kuitunen A, Jukarainen S, Bendel S, et al. Association of endothelial and glycocalyx injury biomarkers with fluid administration, development of acute kidney injury, and 90-day mortality: data from the FINNAKI observational study. Annals of intensive care. 2019;9(1):1-11. |
| 8 | Li X, Sun S, Wu G, Che X, Zhang J. Effect of hydroxyethyl starch loading on glycocalyx shedding and cerebral metabolism during surgery. Journal of Surgical Research. 2020;246:274-83. |
| 9 | Liu Y, Chen G, Gao J, Chi M, Mao M, Shi Y, et al. Effect of different levels of stroke volume variation on the endothelial glycocalyx of patients undergoing colorectal surgery: a randomized clinical trial. Experimental Physiology. 2021;106(10):2124-32. |
| 10 | Macdonald S, Bosio E, Keijzers G, Burrows S, Hibbs M, O’Donoghue H, et al. Effect of intravenous fluid volume on biomarkers of endothelial glycocalyx shedding and inflammation during initial resuscitation of sepsis. Intensive care experimental medicine. 2023;11(1):21. |
| 11 | Macdonald S, Bosio E, Shapiro NI, Balmer L, Burrows S, Hibbs M, et al. No association between intravenous fluid volume and endothelial glycocalyx shedding in patients undergoing resuscitation for sepsis in the emergency department. Scientific reports. 2022;12(1):8733. |
| 12 | Nemme J, Krizhanovskii C, Ntika S, Sabelnikovs O, Vanags I, Hahn RG. Hypervolemia does not cause degradation of the endothelial glycocalyx layer during open hysterectomy performed under sevoflurane or propofol anesthesia. Acta anaesthesiologica scandinavica. 2020;64(4):538-45. |
| 13 | Pouska J, Tegl V, Astapenko D, Cerny V, Lehmann C, Benes J. Impact of intravenous fluid challenge infusion time on macrocirculation and endothelial glycocalyx in surgical and critically ill patients. BioMed research international. 2018;2018. |
| 14 | Powell M, Mathru M, Brandon A, Patel R, Frölich M. Assessment of endothelial glycocalyx disruption in term parturients receiving a fluid bolus before spinal anesthesia: a prospective observational study. International journal of obstetric anesthesia. 2014;23(4):330-4. |
| 15 | Saoraya J, Wongsamita L, Srisawat N, Musikatavorn K. Plasma syndecan-1 is associated with fluid requirements and clinical outcomes in emergency department patients with sepsis. The American Journal of Emergency Medicine. 2021;42:83-9. |
| 16 | Smart L, Macdonald SP, Burrows S, Bosio E, Arendts G, Fatovich DM. Endothelial glycocalyx biomarkers increase in patients with infection during Emergency Department treatment. Journal of critical care. 2017;42:304-9. |
| 17 | Tapking C, Hernekamp J, Horter J, Kneser U, Haug V, Vogelpohl J, et al. Influence of burn severity on endothelial glycocalyx shedding following thermal trauma: a prospective observational study. Burns. 2021;47(3):621-7. |
| 18 | Wang X, Duan Y, Gao Z, Gu J. Effect of Goal-directed Fluid Therapy on the Shedding of the Glycocalyx Layer in Retroperitoneal Tumour Resection. Journal of the College of Physicians and Surgeons--Pakistan: JCPSP. 2021;31(10):1179-85. |
| 19 | Wu X, Hu Z, Yuan H, Chen L, Li Y, Zhao C. Fluid resuscitation and markers of glycocalyx degradation in severe sepsis. Open Medicine. 2017;12(1):409-16. |
| 20 | Smart L, Macdonald SP, Bosio E, Fatovich D, Neil C, Arendts G. Bolus therapy with 3% hypertonic saline or 0.9% saline in emergency department patients with suspected sepsis: a pilot randomised controlled trial. Journal of critical care. 2019;52:33-9. |
